# ﻿*Vacciniumusneoides* (Ericaceae), a new species from Yunnan, China

**DOI:** 10.3897/phytokeys.236.112658

**Published:** 2023-12-21

**Authors:** Yong-Jie Guo, Ting Zhang, Ji-Dong Ya, Wei Zhang, Xiu-Ying Shen, Zhou-Dong Han, Jing-Bo Ni, Jian-Yong Su, Yi-Hua Tong

**Affiliations:** 1 Germplasm Bank of Wild Species, Kunming Institute of Botany, Chinese Academy of Sciences, Kunming, 650201, China; 2 University of Chinese Academy of Sciences, Beijing, 100049, China; 3 Academy of Biodiversity, Southwest Forestry University, Kunming, 650224, Yunnan, China; 4 Fugong Branch of Nujiang Administration of Gaoligongshan National Nature Reserve, Fugong, 673400, Yunnan, China; 5 CAS Key Laboratory for Plant Diversity and Biogeography of East Asia, Kunming Institute of Botany, Chinese Academy of Sciences, Kunming, 650201, China; 6 Key Laboratory of Digital Botanical Garden of Guangdong Province, South China Botanical Garden, Chinese Academy of Sciences, Guangzhou, 510650, China; 7 South China National Botanical Garden, Chinese Academy of Sciences, Guangzhou, 510650, China; 8 Nujiang Administration of Gaoligongshan National Nature Reserve, Lushui, 673100, Yunnan, China; 9 State Key Laboratory of Plant Diversity and Specialty Crops, South China Botanical Garden, Chinese Academy of Sciences, Guangzhou, 510650, China

**Keywords:** Gaoligong Mountain, morphology, Vaccinieae, *Vacciniumarbutoides*

## Abstract

*Vacciniumusneoides* (Ericaceae), a new species from Fugong County of Yunnan Province, China is described and illustrated. This new species belongs to Vacciniumsect.Calcicolus and is most similar to *V.brachyandrum*, but differs in its branches hanging down, much smaller leaf blades, shorter inflorescences and pedicels, non-glandular tomentellate or densely pubescent inflorescence rachis and pedicels, densely white-pubescent hypanthium and pilose filaments.

## ﻿Introduction

The genus *Vaccinium* L. (Ericaceae), with about 450–500 species distributed worldwide, is the largest genus of the blueberry tribe (Fang 1991; Fang and Stevens 2005; Vander Kloet and Dickinson 2009). This genus is a morphologically diverse group, which was divided into 33 (Sleumer 1941) or 30 sections (Vander Kloet and Dickinson 2009) with most sections of this genus allopatric. In China, 100 species of *Vaccinium* have been recorded, including the very recently published *V.jiuwanshanense* Ying Qin, Yan Liu & Y. H. Tong and *Vacciniumbangliangense* Y. S. Huang & Y. H. Tong (Huang et al. 2022; Qin et al. 2023). Yunnan Province, with 47 species of *Vaccinium*, harbours the highest diversity of this genus in China (Fang 1986; Huang and Fang 1991).

During several field trips to Gaoligong Mountain, we encountered an interesting *Vaccinium* species. This species is epiphytic on large trees with long hanging-down branches, which looks just like beard lichens from a distance. The inflorescence of this species is very hairy and shorter than and shaded by leaf blades, suggesting a close relationship with *V.brachyandrum* C. Y. Wu & R. C. Fang, another sympatrically distributed species in Gaoligong Mountain. However, *V.brachyandrum* owns scrambling branches and much larger leaf blades, which are totally different from this unknown species. After a careful comparison of morphology with similar congeneric species from China and adjacent Myanmar (Kress et al. 2003; Fang and Stevens 2005), it was confirmed that this plant represents a new species, which is described and illustrated below.

## ﻿Material and methods

Flowering and fruiting materials were collected from Gaoligong Mountain during three field trips in May 2022, October 2022 and March 2023. Descriptions were based on both living and dried collections. The voucher specimens were deposited at the Herbaria of Kunming Institute of Botany, Chinese Academy of Sciences (KUN) and South China Botanical Garden, Chinese Academy of Sciences (IBSC). Measurements were performed with a ruler and small plant parts were observed and measured under a stereomicroscope (Mshot-MZ101).

## ﻿Taxonomic treatment

### ﻿ Vaccinium usneoides

Taxon classificationPlantaeEricalesEricaceae

Y.H.Tong, Y.J.Guo & Ting Zhang sp. nov.

8BD180ED-2D48-51B2-84F7-44036A77A207

urn:lsid:ipni.org:names:77333175-1

Figs 1 , 2

#### Type.

China. Yunnan Province: Fugong County, Shiyueliang Xiang, Yaduo Village, Nihajiadi (also called “Shibagongli” unofficially), Gaoligong Mountain, epiphytic on trees in evergreen and deciduous broad-leaved mixed forest, 27°9′55.0″N, 98°46′44.2″E, 2497 m a.s.l., 27 May 2022 (fl.), *Ting Zhang, Ji-Dong Ya & Wei Zhang 22CS21979* (holotype: KUN, barcode no. 1584163 (Fig. 3), isotypes: IBSC, barcode no. 1003856, KUN, barcode no. 1584164).

#### Diagnosis.

This new species is close to *V.brachyandrum* in the short and hairy inflorescences (less than 3.5 cm) with many flowers and the abaxially glandular leaf blades with one basal gland per side and a caudate-acuminate apex, but can be immediately distinguished by its hanging-down (vs. scrambling) branches, much smaller leaf blades (2.5–5.5 × 0.9–1.8 cm vs. 8.5–11 × 4–6 cm) with fewer pairs of secondary veins (3–4 vs. 6–7), shorter inflorescence (1–1.5 cm vs. 1.5–3.5 cm), non-glandular tomentellate or densely pubescent (vs. glandular pubescent) inflorescence rachis and pedicel, shorter pedicel (0.7–1 mm vs. ca. 2 mm), densely white-pubescent (vs. glabrous) hypanthium and pilose (vs. glabrous) filament. A detailed morphological comparison between the two species is presented in Table 1.

**Figure 1.**
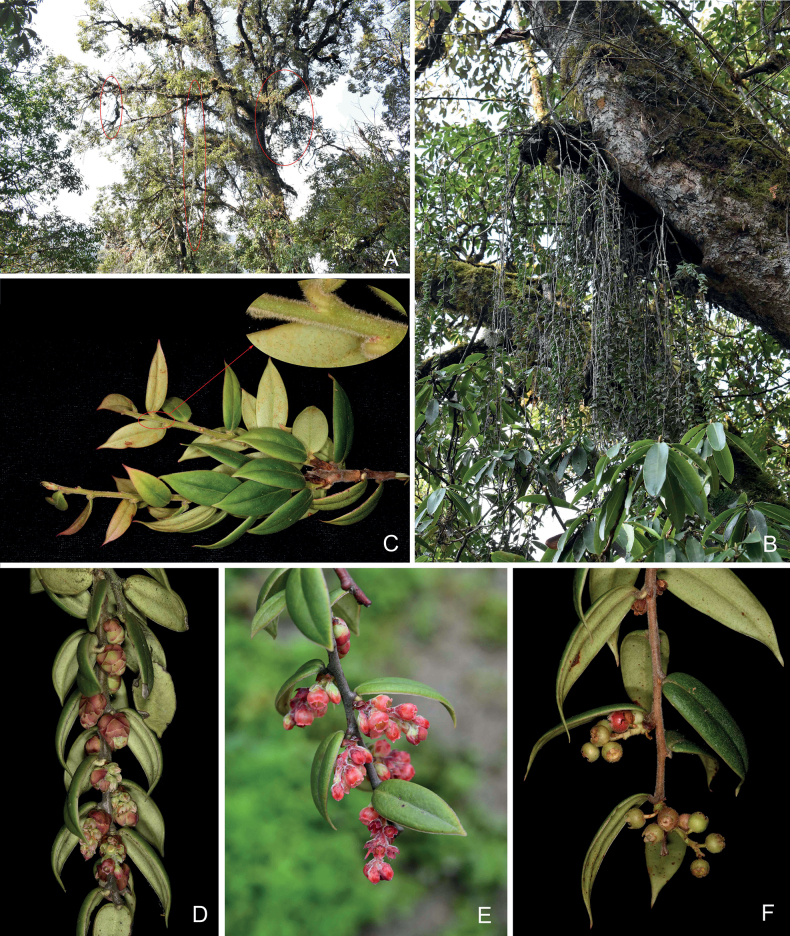
*Vacciniumusneoides* sp. nov. **A** habitat, the red ovals indicating this species **B** habit **C** leafy branches, the arrow showing the indumentum on young branchlets and leaf blades **D** flowering branchlets with young inflorescences **E** flowering branchlet **F** fruiting branchlet. **A, B** taken by Yi-Hua Tong **C, F** taken by Yong-jie Guo **D** taken by Ji-Dong Ya **E** taken by Ting Zhang.

**Table 1.** A morphological comparison of *Vacciniumusneoides* and *V.brachyandrum*. The character information of the latter species is taken from Fang and Wu (1987), Fang and Stevens (2005) and its type specimens.

Characters	*V.usneoides*	*V.brachyandrum*
Branches	Hanging down	Scrambling
Leaf blade	2.5–5.5 × 0.9–1.8 cm	8.5–11 × 4–6 cm
Pairs of secondary veins	3–4	6–7
Inflorescence length	1–1.5 cm	1.5–3.5 cm
Indumentum on inflorescence and pedicel	Non-glandular tomentellate or densely pubescent	Glandular pubescent
Hypanthium	Densely white-pubescent	Glabrous
Pedicel length	0.7–1 mm	ca. 2 mm
Filament	Pilose	Glabrous

#### Description.

Evergreen shrubs, epiphytic on tree trunks, sparsely branched; roots creeping firmly on tree trunks or branches; stems 0.5–3 m long, without swollen basal tuber or root swellings. Branches hanging down, young shoots brownish or greenish, terete or slightly angled, without lenticels, densely pubescent; old ones more or less glabrescent, brownish or greyish, often obviously angled in sicco. Perennating buds dimorphic (floral perennating buds at least twice the size of vegetative perennating buds). Leaves alternate; petiole short, curved, 2–5 mm long, 1–1.5 mm wide, densely pubescent, glabrescent when mature; blade narrowly ovate to oblong, 2.5–5.5 (including caudate apex) × 0.9–1.8 cm, thickly leathery, adaxially more or less transversely wrinkled when dry, especially for young ones, abaxially with evenly distributed and caducous appressed black glandular trichomes, trichome base papillate, both sides pubescent when young, glabrous when mature, base rounded to broadly cuneate, with 1 basal gland per side at ca. 1 mm distance from the junction of leaf blade base and petiole, margin entire, revolute, with a ca. 0.5 mm broad, cartilaginous edge, apex caudate-acuminate; veins impressed adaxially, more so when dry, obscure abaxially, secondary veins 3–4 per side, tertiary veins slightly prominent. Inflorescence short racemose, 1–1.5 cm long, axillary on biennial branches, shorter than and shaded by leaf blades, 8–14-flowered. Peduncle very short, inflorescence rachis 0.7–1 cm long, white-tomentellate or densely white-pubescent; bracts red on exposed part, greenish on covered part, broadly obovate, cucullate, 5–6 × 6–7 mm, abaxially more or less white-pubescent on mid-vein, more so on lower half, adaxially glabrous, margin ciliate, caducous; bracteoles 2, inserted at base of pedicel, greenish, sometimes tinged with red, obovate, 3.5–4 × 2–2.5 mm, indumentum similar to bracts, caducous. Pedicels greenish, very short, 0.5–1 mm long, densely white-pubescent, articulate with the hypanthium. Hypanthium greenish, obconical, 1–1.5 × 1–1.5 mm, densely white-pubescent, more so at base; calyx limb lobed nearly to base, lobes 5, green, more or less tinged with red, triangular, ca. 2 × 1.5 mm, abaxially white-pubescent at apex, otherwise glabrous, adaxially glabrous, apex acuminate, margin ciliate. Corolla red, urceolate-campanulate, slightly angled when young, 3.5–4 × ca. 3 mm, tube glabrous on both sides; lobes 5, ovate-triangular, reflexed, ca. 0.8 × 1 mm, abaxially white-pubescent at apex, otherwise glabrous, adaxially papillose, apex acuminate. Stamens 10, 2.8–3 mm long; filaments flat, slightly S-shaped, 1–1.2 mm long, papillose on both sides, margin pilose; anthers 1.8–2 mm long, thecae slightly longer than or nearly as equal as tubules; spurs 2, borne at abaxial base of tubules, 0.7–0.8 mm long, spreading. Disc yellowish-green, annular, glabrous; style cylindrical, slightly angled in sicco, 2.7–3 mm long, glabrous, stigma truncate; ovary pseudo-10-locular, each locule with several ovules. Fruiting pedicels 1–3.5 mm long; berries pale green to green when young, yellowish-green to purplish-red or dark purple when mature, globose, 4.5–6 mm in diam., densely white-pubescent, with persistent calyx lobes appressed at apex, ripe berries a little bitter. Seeds ovoid, 0.7–0.8 × 0.5–0.7 mm, testa brownish, reticulate.

**Figure 2.**
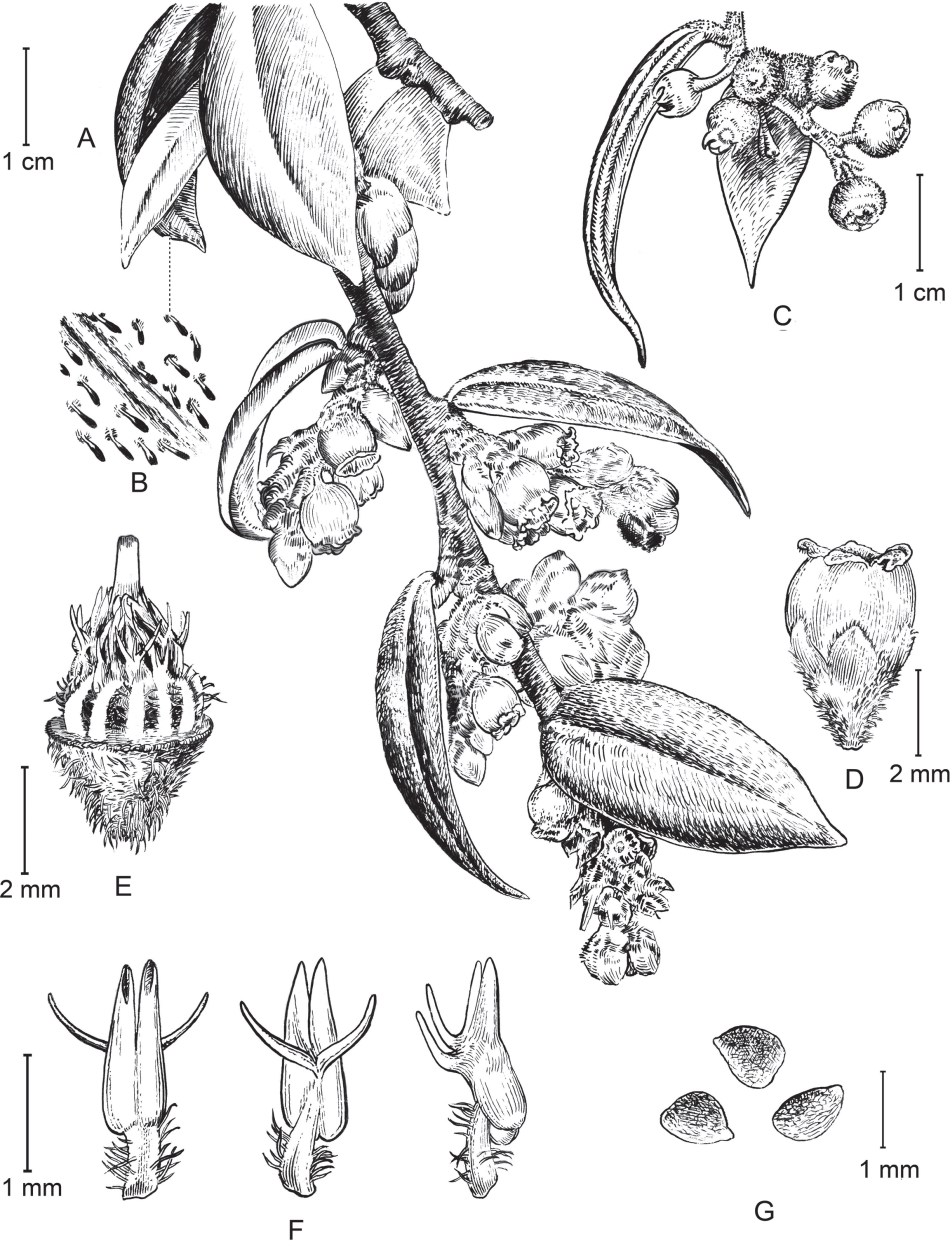
*Vacciniumusneoides* sp. nov. **A** flowering branchlet **B** trichomes on abaxial surface of leaf blades **C** fruiting branchlet **D** flower **E** flower with corolla and calyx limb removed, showing androecium and gynoecium **F** stamens, adaxial (left), abaxial (middle) and lateral (right) view **G** seeds. Illustrated by Ding-Han Cui.

**Figure 3.**
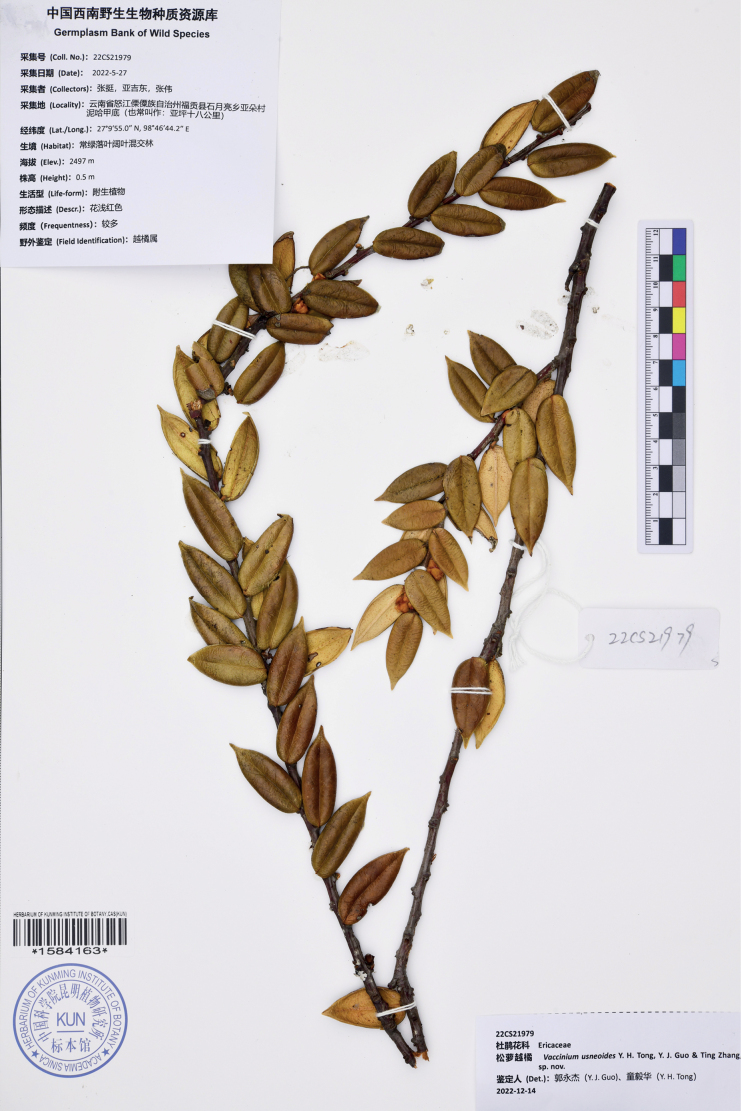
Holotype of *Vacciniumusneoides* (*Ting Zhang, Ji-Dong Ya & Wei Zhang 22CS21979*, KUN, barcode no. 1584163).

#### Etymology.

The species epithet is derived from the genus of beard lichen “*Usnea*” and the suffix “-*oides*”, which means the habit of this new species looks very much like beard lichen on trees from a distance. Chinese name is given as松萝越橘 (Pinyin: sōng luó yuè jú).

#### Distribution and habitat.

This species is currently known only from the type locality, i.e. the part of the Gaoligong Mountain in Fugong County. It grows on trees in mountainous evergreen and deciduous broad-leaved mixed forests at elevations of 2400–2800 m a.s.l.

#### Conservation status.

*Vacciniumusneoides* is not rare in the type locality and the whole distribution area is under the protection of Gaoligongshan National Nature Reserve. Thus, the threat risk seems to be low. Since the type locality is very near the border of China and Myanmar, this species is probably also distributed in the adjacent area of Myanmar. Thus, it is best to assign a status of ‘Data Deficient’ (DD) for this species following the IUCN Red List Categories and Criteria (IUCN Standards and Petitions Committee 2022).

#### Phenology.

Flowering in May–June and fruiting in October–November.

#### Additional specimens examined.

*Vacciniumusneoides* (paratypes): the same locality with holotype: 27°9′57.78″N, 98°46′14.91″E, 2661 m a.s.l., 27 October 2022 (fr.), *Yong-Jie Guo, Zhou-Dong Han & Xiu-Ying Shen 22CS22598* (IBSC, barcode no. 1003853, KUN, barcode nos. 1584165 & 1584166); 27°9′56.89″N, 98°46′14.63″E, 2652 m a.s.l., 27 October 2022 (fr.), *Yong-Jie Guo, Zhou-Dong Han & Xiu-Ying Shen 22CS24002* (IBSC, barcode no. 1003856, KUN, barcode nos. 1584167 & 1584168); 27°9′46.08″N, 98°46′39.98″E, 2544 m a.s.l., 27 October 2022 (fr.), *Yong-Jie Guo, Zhou-Dong Han & Xiu-Ying Shen 22CS24003* (IBSC, barcode no. 1003855, KUN, barcode no. 1584169); 27°9′39.6″N, 98°46′56.06″E, 2444 m a.s.l., 10 March 2023, *Yi-Hua Tong, Jing-Bo Ni, Bing-Mou Wang & Wei-Hao Pan TYH-2637* (IBSC).

#### Examined specimens of *Vacciniumbrachyandrum*.

China. Yunnan Province: Tengchong, Houqiao, 2720 m a.s.l., 18 May 1964, *Su Kung Wu 6629* (holotype KUN1209465, isotype KUN1209466); ibid., 25°24′16.700″N, 98°8′43.869″E, 2564 m a.s.l., 22 August 2022, *Yong-Jie Guo & Zhong-Lan Yang 22CS22523* (IBSC, KUN); Lushui, Pianma, Fengxueyakou, 25°59′12.779″N, 98°40′3.828″E, 2691 m a.s.l., 23 May 2022, *Ting Zhang, Ji-Dong Ya & Wei Zhang 22CS21913* (IBSC, KUN); ibid., 25°59′12.72″N, 98°40′3.9″E, 2705 m a.s.l., 28 October 2022, *Yong-Jie Guo & Zhou-Dong Han 22CS22599* (IBSC, KUN); ibid., 25°59′0.76″N, 98°40′5.03″E, 2782 m a.s.l., 11 March 2023, *Yi-Hua Tong, Jing-Bo Ni, Bing-Mou Wang & Wei-Hao Pan TYH-2643* (IBSC) & *TYH-2644* (IBSC).

## ﻿Discussion

In the key to *Vaccinium* in “Flora of China” (Fang and Stevens 2005), *V.usneoides* is keyed out to be close to *V.arbutoides* C. B. Clarke and *V.brachyandrum* (item 47). However, we found that the description of *V.arbutoides* in “Flora of China” is inconsistent with that in the protologue (although the description made by Clarke (1881–1882) is brief) on some important characters, such as the length of petiole (6–9 mm in “Flora of China” vs. 3.2–5.1 mm in the protologue, the same order for the following comparison), inflorescence (1.5–3 cm vs. 6.35 cm), pedicel (1–3 mm vs. 4.2 mm) and calyx lobe (ca. 2.5 mm vs. 3.2 mm), which made us suspect that the Chinese material of this species (the only one collection, *Tse-Tsun Yu 20963* (SZ) from Gongshan County, Yunnan Province) may be misidentified. However, due to incomplete information on floral morphology of the type collection (*Griffith 3469* (K) from northeast India) and the Chinese material, the relationship between these two collections remains uncertain, whether they belong to the same species or not needs further study. Nonetheless, compared to the large leaf blades (ca. 12.7 × 6.35 cm) of *V.arbutoides* (Clarke 1881–1882), the leaf blades of *V.usneoides* are so much smaller (2.5–5.5 × 0.9–1.8 cm) that they can hardly be recognised as conspecifics.

According to Vander Kloet and Dickinson’s infrageneric classification of *Vaccinium*, *V.usneoides* fits well with the circumscription of V.sect.Calcicolus Kloet, which is characterised by an evergreen habit, dimorphic perennating buds (i.e. floral perennating buds at least twice the size of vegetative perennating buds), racemose inflorescences with large caducous bracts, pseudo-10-locular ovary, berry with 2–5 seeds per locule and soft seed testa (Vander Kloet and Dickinson 2005, 2009). This new species usually grows on large trees (e.g. *Acercampbellii* Hook. f. & Thomson ex Hiern, *Quercussaravanensis* A. Camus etc.) trunks covered with mosses, orchids, ferns and some other ericaceous species like *Agapetespensilis* Airy Shaw, *Vacciniumleucobotrys* (Nutt. ex Hook.) G. Nicholson and *V.dendrocharis* Hand.-Mazz.

## Supplementary Material

XML Treatment for Vaccinium usneoides

## ﻿Additional information

### Author ORCIDs

Yong-Jie Guo https://orcid.org/0000-0003-1752-4495

Ting Zhang https://orcid.org/0000-0003-0939-8468

Ji-Dong Ya https://orcid.org/0000-0003-3389-1412

Wei Zhang https://orcid.org/0009-0003-5327-8698

Zhou-Dong Han https://orcid.org/0009-0001-9431-1349

Yi-Hua Tong https://orcid.org/0000-0002-5034-005X

### Data availability

All of the data that support the findings of this study are available in the main text.
